# Evaluation of Self-care Activities and Quality of Life in Patients With Type 2 Diabetes Treated With Metformin Using the 2D Matrix Code of Outer Drug Packages as Patient Identifier: the DePRO Proof-of-Concept Observational Study

**DOI:** 10.2196/31832

**Published:** 2022-05-24

**Authors:** Christian Mueller, Isabel Schauerte, Stephan Martin, Valeska Irrgang

**Affiliations:** 1 Bayer Vital GmbH Leverkusen Germany; 2 Institut Dr. Schauerte Munich Germany; 3 Westdeutsches Diabetes- und Gesundheitszentrum Düsseldorf Germany

**Keywords:** self-care activities, quality of life, diabetes mellitus, type 2, patient reported outcome, PRO, digital observational study, bring your own device, BYOD, diabetes, diabetes self management, digital health, patient reported outcomes, virtual care, health application, mHealth, mobile health

## Abstract

**Background:**

The use of digital technology to assess patients remotely can reduce clinical study costs. In the European Union, the 2D matrix code on prescription drug packaging serves as a unique identifier of a given package of medication, and thus, also of the patient receiving that medication. Scanning of the 2D matrix code may therefore allow remote patient authentication in clinical studies.

**Objective:**

The aim of the DePRO study was to assess the feasibility of a fully digital data-capture workflow, the authentication of participants via drug packaging 2D matrix codes, in patients with type 2 diabetes mellitus (T2DM) who use metformin. The primary objective was to describe the self-care activities of these patients. Secondary objectives were to evaluate (1) the self-reported health status of these patients, (2) the association of self-care activities with demographics and disease characteristics, and (3) the usability of the my ePRO app.

**Methods:**

DePRO was an observational, multicenter, cross-sectional, digital, and patient-driven study conducted in Germany from June to December 2020. Adult patients prescribed metformin were invited to participate via their pharmacist or a medication tracker app. Participants downloaded the my ePRO app onto their own mobile device, scanned the 2D matrix code on their metformin package for registration and authentication, and provided informed consent via an electronic form. They were then able to complete a study-specific questionnaire on demographics and clinical characteristics, the German version of the Summary of Diabetes Self-Care Activities measure (SDSCA-G), the Diabetes Treatment Satisfaction Questionnaire (DTSQ), and the EQ-5D-5L. The patients conducted the study without support from a health care professional. Statistical analyses were exploratory and descriptive.

**Results:**

In total, 3219 patients were invited to participate. The proportion of patients giving consent was greater among those invited by pharmacists (19/217, 8.8%) than among those invited via the medication tracker app (13/3002, 0.4%). Of the 29 patients eligible for analysis, 28 (97%) completed all study questionnaires. Most of the patients (23/29, 79%) were aged <60 years, and 59% (17/29) were male. The patients spent a mean total of 3.5 (SD 1.3) days out of 7 days on self-care activities (SDSCA-G). Most patients (24/29, 83%) were satisfied to extremely satisfied with their current treatment (DTSQ). Events of perceived hyperglycemia or hypoglycemia were reported by 20 of 29 (69%) patients. The best possible health status (EQ-5D-5L) was reported by 18 of 28 (64%) patients. Age was positively correlated with time spent on general and specific diet (Spearman coefficient 0.390 and 0.434, respectively).

**Conclusions:**

The DePRO study demonstrates the feasibility of fully digital authentication (via 2D matrix codes on drug packaging) and data capture in patients with T2DM. Personal invitations yielded higher recruitment rates than remote invitations via the medication tracker app. A high questionnaire completion rate was realized, based on completion by 28 out of 29 patients.

**Trial Registration:**

ClinicalTrials.gov NCT04383041; https://clinicaltrials.gov/ct2/show/NCT04383041

**International Registered Report Identifier (IRRID):**

RR2-10.2196/21727

## Introduction

In clinical studies, the need for participants to visit a study clinic for on-site assessments is an important driver of cost [1]. The use of digital technology for remote assessment of study participants can remove the need for site visits, as exemplified by several recent clinical studies without study sites [2-4].

In the European Union, all prescription drugs (with specific exceptions such as radionuclide generators and precursors) are required to bear a 2D matrix code on their packaging [5]. The 2D matrix code is a unique identifier of a given package of medication; once the medication is given to a patient, the code also serves as a unique identifier of the patient as the user of that medication. Scanning of the 2D matrix code therefore has the potential to allow remote patient authentication across a range of indications.

We designed the DePRO study to assess the feasibility of a fully digital data-capture workflow with the authentication of eligible patients via the 2D matrix codes on drug packaging [6]. The target population was patients with type 2 diabetes mellitus (T2DM) who had been prescribed metformin (the recommended initial treatment for T2DM [7]). For patients with T2DM, clinical recommendations suggest a key role for self-care in maintaining health and quality of life [8,9]. Therefore, the primary objective of the DePRO study was to describe the self-care activities of patients with T2DM who use metformin. Secondary objectives were to evaluate (1) the self-reported health status of these patients, (2) the association of self-care activities with demographics and disease characteristics, and (3) the usability of the my ePRO app (acceptance of participation and completion of study questionnaires).

## Methods

### Study Design

The study design has been published previously [6] and is briefly summarized here.

DePRO was an observational, multicenter, cross-sectional, digital, and patient-driven study conducted in Germany from June 2020 to December 2020 (ClinicalTrials.gov NCT04383041). The study was conducted using the my ePRO app, a patient-reported outcome (PRO) data capture tool that can gather additional data based on individual study requirements (codeveloped by Institut Dr. Schauerte and Bayer and hosted by Institut Dr. Schauerte). The my ePRO app is not a self-care management tool supporting patients in their treatment of T2DM.

Adult patients who had been prescribed metformin-containing medications were eligible to participate. Eligible patients were invited via their pharmacist (who personally handed out a download link for the my ePRO app on a postcard alongside the patient’s metformin-containing medication and explained the study workflow to the patients—downloading the my ePRO app, scanning the 2D matrix code, consenting, answering the questionnaires, and then getting reimbursed) or via the MyTherapy medication tracker app (SmartPatient). The latter route was added as a revision to the original published study design [6] following difficulties recruiting sufficient pharmacies because of restrictions related to the COVID-19 pandemic (we originally planned to recruit 12 diabetes-focused pharmacies, but of the 35 pharmacies contacted, only 4 participated in the study). All patients who were registered on the medication tracker app and were taking metformin-containing medications in Germany were invited twice (on December 2 and 7, 2020) via an in-app message to participate in the DePRO study. All patients who were taking metformin received the invitation (which was automatically deployed) in their daily to-do list and voluntarily downloaded the my ePRO app to participate in the DePRO study. The recruitment finished on December 9, 2020. The medication tracker app provided a download link for the my ePRO app.

In each case, participating patients downloaded the my ePRO app onto their own mobile device (smartphone or tablet). They then scanned the unique 2D matrix code on their metformin-containing medication package for registration and authentication. The 2D matrix code includes an identifier of the drug package, which is unique, and the pharma central number of the drug. The sponsors of the study were only aware of which 2D matrix codes were used and could verify eligibility of the patients via the pharma central number. By using the my ePRO app, no additional personal data were requested for participation, ensuring the anonymity and privacy of all participants. After providing informed consent via an electronic form, they were able to complete a study-specific questionnaire on patient characteristics and 3 validated questionnaires: the German version of the Summary of Diabetes Self-Care Activities measure (SDSCA-G) [10]; the Diabetes Treatment Satisfaction Questionnaire (DTSQ; possible score range of 0-36, with higher scores reflecting greater satisfaction) [11]; and the EQ-5D-5L [12]. After completing the questionnaires, each patient received compensation (a voucher worth €15 [US $15.82]) for the time spent providing data. The patients conducted the entire study without any support from their pharmacist or health care professional.

Data were transferred to the study database upon completion of each questionnaire and as soon as the patient’s device was connected to the internet. Reports of hyperglycemia and hypoglycemia were identified by the contract research organization (Institut Dr. Schauerte) and forwarded as potential adverse events to the relevant market authorization holders.

### Statistical Analysis

Statistical analyses were exploratory, descriptive, and performed using SAS statistical software (version 9.4 or higher; SAS Institute Inc). Spearman rank correlation coefficients were used as measures of association, with *P*<.05 considered significant. We estimated that a sample size of 300 patients would be required to obtain a 95% CI of the mean level of self-care with a precision of 3 points [6].

### Ethics Approval

The study protocol has been approved by the Ethics Committee of the Medical Association Nordrhein (approval number 2020084).

## Results

### Patients

**Figure 1:**
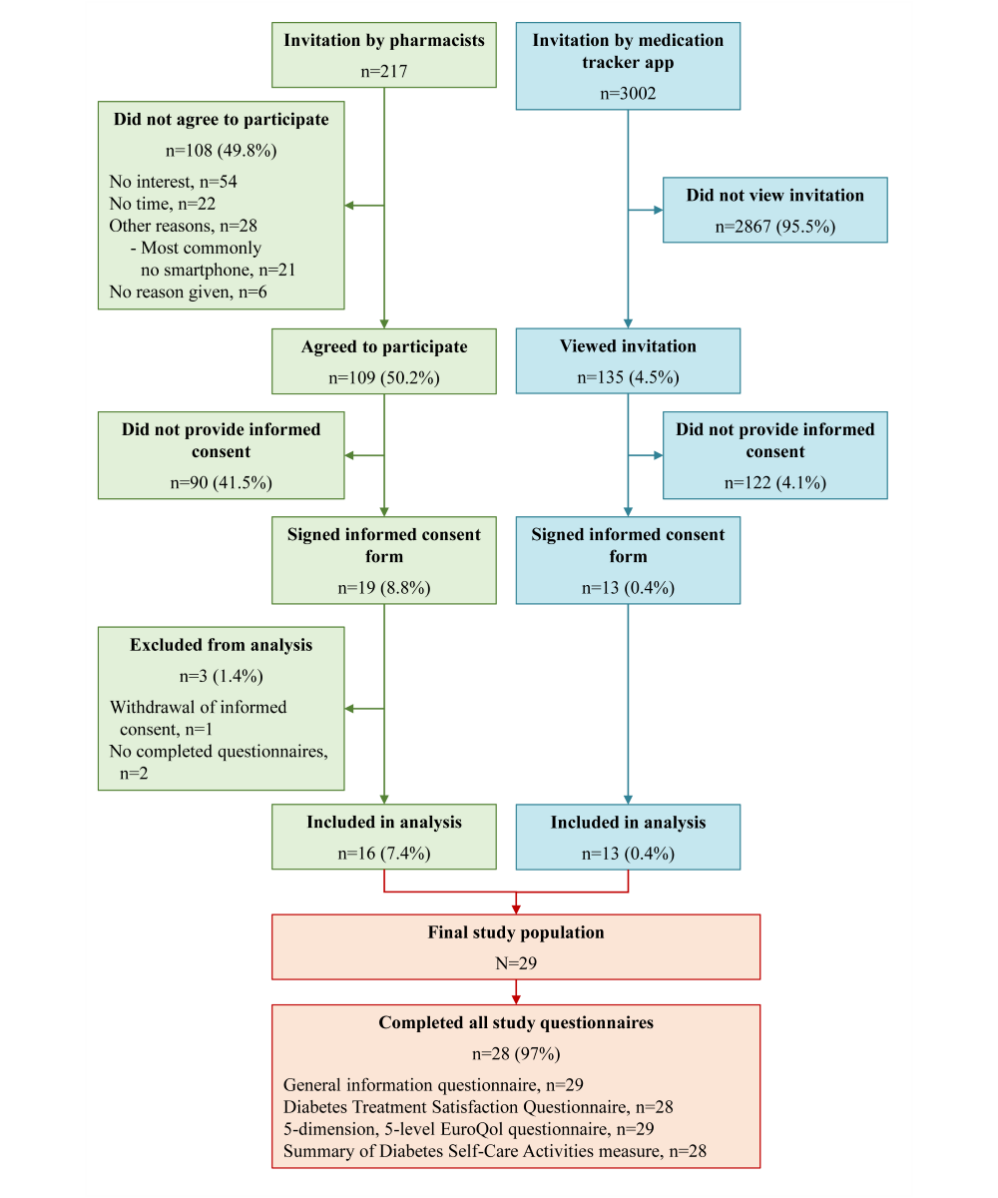
Flow chart of study participation. An individual patient could give multiple reasons for not agreeing to participate.

A total of 3219 patients were invited to participate in the study, either by their pharmacist (n=217) or via the medication tracker app (n=3002; Figure 1). Of the 217 patients invited by their pharmacist, 108 did not agree to participate, most commonly because they had no interest (n=54), no time (n=22), or no smartphone (n=21). The proportion of patients who gave consent was greater among those invited by their pharmacist (19/217, 8.8%) than among those invited via the medication tracker app (13/3002, 0.4%). However, 3 patients were excluded due to the withdrawal of informed consent or because they did not complete at least one questionnaire. Thus, 29 patients were eligible for analysis. Of the 29 patients, 28 (97%) completed all study questionnaires; 16 (55%) patients answered all of the questions, 12 (41%) answered ≥80% to <100% of the questions, and 1 (3%) answered <80% of the questions.

Patient characteristics are summarized in Table 1. In total, 59% (17/29) of the patients were male and 79% (23/29) were aged <60 years. A substantial proportion (9/29, 31%) did not report their family income (this was also observed during user-experience testing of the my ePRO app). According to their zip codes, the majority of the patients (17/29, 59%) lived in western Germany, reflecting the location of the participating pharmacies. Concomitant medication use was reported by 12/29 (41%) patients; the most commonly reported concomitant medications were those acting on the cardiovascular system (9/29, 31%).

Most of the patients (27/29, 93%) reported having visited an ophthalmologist, but few reported eye-related comorbidities: 1 patient reported that their ophthalmologist had seen changes in their retina/fundus caused by diabetes, and another patient had received laser eye surgery or intraocular medication injections. Responses to other questions regarding comorbidities are summarized in Multimedia Appendix 1.

**Table 1:** Patient characteristics.

Characteristic			Patients, n (%)
Total study population			29 (100)
**Gender**			
	Female	12 (41)	
	Male	17 (59)	
**Age (years)**			
	<40	10 (34)	
	40-59	13 (45)	
	≥60	6 (21)	
**BMI (kg/m^2^)**			
	<25	3 (10)	
	25 to <30	12 (41)	
	30 to <35	7 (24)	
	≥35	7 (24)	
**Education level**			
	Not reported	1 (3)	
	No certificate	3 (10)	
	General secondary school	3 (10)	
	Vocational education	13 (45)	
	Intermediate secondary school	2 (7)	
	High school	1 (3)	
	College or university	6 (21)	
**Family income (€ gross/month; US$ gross/month)**			
	Not reported	9 (31)	
	2000 to <3000 (2108 to <3162)	10 (34)	
	3000 to <5000 (3162 to <5270)	8 (28)	
	≥5000 (≥5270)	2 (7)	
**Geographic region in Germany**			
	North	3 (10)	
	East	4 (14)	
	South	5 (17)	
	West	17 (59)	
**Time since diagnosis of type 2 diabetes mellitus (years)**			
	Not reported	2 (7)	
	<1	5 (17)	
	1-5	10 (34)	
	6-10	8 (28)	
	≥11	4 (14)	
**Latest glycated hemoglobin value (%)**			
	Missing	2 (7)	
	<6.0	11 (38)	
	6.0-6.5	3 (10)	
	6.6-7.0	4 (14)	
	7.1-7.5	4 (14)	
	7.6-8.0	0	
	8.1-8.5	3 (10)	
	8.6-9.0	1 (3)	
	>9.0	1 (3)	
**Concomitant medications**			
	0	17 (59)	
	1	5 (17)	
	2	4 (14)	
	3	1 (3)	
	4	1 (3)	
	6	1 (3)	

### Self-care Activities, Quality of Life, and Treatment Satisfaction

According to the SDSCA-G, the patients spent a mean total of 3.5 (SD 1.3) days out of 7 days on self-care activities. The greatest mean number of days was spent on general diet (4.7, SD 1.9 days), followed by specific diet (3.9, SD 2.9 days), exercise (3.8, SD 2.1 days), and blood-glucose testing (3.4, SD 2.6 days). Data were missing for 1 patient in each sub-score. A total of 8/29 (28%) patients reported that they smoked.

The participants had a median EQ-5D-5L index score of 1.00 (IQR 0.73-1.00; n=28). The best possible health status was reported by 18/28 (64%) patients. The median EuroQol visual analog scale score was 79.0 (IQR 49.6-99.0; n=29). The majority of the patients reported no problems with mobility, self-care, usual activity, or anxiety and depression, whereas more than half (15/29, 52%) reported problems with pain and discomfort (Table 2).

The median DTSQ score was 24.5 (IQR 12.0-30.0; n=28). Most patients (24/29, 83%) were satisfied to extremely satisfied with their current treatment (Multimedia Appendix 2).

Overall, 20/29 (69%) patients reported events of perceived hyperglycemia or hypoglycemia. High scores (≥4) for perceived hyperglycemia and hypoglycemia were reported by few patients (4/29, 14% and 2/29, 7%, respectively).

Age was positively correlated with time spent on general diet (Spearman coefficient 0.390; *P*=.04) and specific diet (Spearman coefficient 0.434; *P*=.02), but not with the total SDSCA-G score or other SDSCA-G subscores (n=28).

**Table 2:** Responses to the 5-dimension, 5-level EuroQol questionnaire.

	Mobility, n (%)	Self-care, n (%)	Usual activity, n (%)	Pain and discomfort, n (%)	Anxiety and depression, n (%)
Total	29 (100)	29 (100)	29 (100)	29 (100)	29 (100)
Missing data	1 (3)	1 (3)	1 (3)	1 (3)	1 (3)
Extreme problems	0 (0)	0 (0)	0 (0)	0 (0)	0 (0)
Severe problems	0 (0)	0 (0)	0 (0)	2 (7)	0 (0)
Moderate problems	2 (7)	0 (0)	1 (3)	4 (14)	4 (14)
Slight problems	1 (3)	2 (7)	4 (14)	9 (31)	5 (17)
No problems	25 (86)	26 (90)	23 (79)	13 (45)	19 (66)

## Discussion

### Principal Findings

The innovative approach taken in the DePRO study was to implement a fully digital data-capture workflow, bypassing the involvement of health care professionals in the assessment of PROs and more importantly in the authentication of eligible patients. Overall, the results demonstrate the feasibility of this approach; participating patients were able to follow the study workflow to completion. We observed a higher rate of enrollment among patients invited by pharmacists than among those invited via the medication tracker app. Although enrollment in the DePRO study was low overall (32/3219, 1%), the proportion of participating patients completing all study questionnaires was high (28/29, 97%). The high rate of completion may be due to the iterative development of the app, which included user-experience testing in patients with T2DM before launching the app.

The patients spent a mean total of 3.5 (SD 1.3) days on self-care activities, and most (24/29, 83%) were satisfied to extremely satisfied with their current treatment. The majority of the patients reported the best health status in the EQ-5D-5L. Positive correlations were found between age and time spent on diet, but the small sample size limits the interpretation of these findings.

### Comparison With Prior Work

The low participation rate among patients invited remotely via the medication tracker app in the DePRO study (13/3002, 0.4%) was broadly similar to that observed in the Apple Heart Study (419,297 patients recruited from among more than 30 million device users, ~1.4%) [2]. The higher rate of enrollment among patients invited by their pharmacist compared to patients invited remotely is consistent with a recent meta-analysis, which found higher conversion rates for offline versus online recruitment strategies (risk ratio 0.8, 95% CI 0.67-0.96; *P*=.02) [13].

Our results suggest that personal contact with a trusted health care professional was an important factor for enrollment in the DePRO study. This personal contact may have helped to overcome potential barriers such as patients’ lack of familiarity with the my ePRO app and concerns regarding data protection. Other possible reasons for the low response rate among patients invited remotely could include the COVID-19 pandemic, the timing of the invitations (December is likely to have been a busy month for many patients), or the media switch—from offline to online or between different systems (from the medication tracker app to the my ePRO app). Altering the invitation design and wording and sending multiple reminders may increase the rate of remote enrollment in future studies, as shown in the mHealth Screening to Prevent Strokes trial [14]. For both invitation routes, the design of the electronic informed consent form may also influence enrollment; we found that a substantial proportion of patients who viewed the remote invitation or agreed to participate when invited by their pharmacist did not provide informed consent.

Many of the patients were using concomitant cardiovascular medications and had visited an ophthalmologist, as expected for a population with T2DM [15-17]. The DePRO study population was relatively young—79% of the study population was aged <60 years; in contrast, only 19.5% of the 324,708 patients with T2DM in a recent German claims database analysis were aged <60 years [15]. Again, the small sample size of the DePRO study limits the interpretation of this finding; larger studies are needed to determine if a fully digital workflow results in a selection bias toward patients who are young technophiles.

The PROs were generally consistent with previous reports. For example, the mean time spent on self-care activities was similar to that reported by 315 patients with T2DM (3.5, SD 1.4 days) in a validation study of the SDSCA-G [10]. In a large survey in Germany (N=1291), patients with T2DM reported greater problems with pain and discomfort than with other EQ-5D-5L dimensions [18], similar to the pattern observed in our study. We found a high rate of treatment satisfaction; this was also shown in a study of 602 patients receiving metformin in Italy where the mean DTSQ scores reflected satisfaction with the treatment overall [19].

### Limitations

The limitations of the DePRO study design have been described in detail previously [6]. Briefly, they include its reliance on PROs, the identification of patients via a 2D matrix code on drug packaging rather than a validated diagnosis by a health care professional, possible selection bias toward patients who are young technophiles, the lack of verification of medication intake, the single-arm design, the focus on users of metformin rather than all patients with T2DM, geographic bias toward western Germany, and anonymized data (making source-data verification impossible).

The small sample size is an additional limitation, which can be explained by a lack of interest in studies and a fear of infection with COVID-19 by staying too long in a pharmacy. Additionally, further meaningful correlations between self-care activities and demographics could not be established due to the limited sample size. We were unable to recruit sufficient pharmacies because of the COVID-19 pandemic, and recruitment via a medication tracker app yielded a low response rate. Nevertheless, the study demonstrates the feasibility of recruiting patients via both pharmacist- and app-based approaches. The app-based approach was implemented rapidly in response to changing circumstances and offers a promising starting point for further development. The optimal recruitment strategy may differ across indications and age groups.

### Conclusions

The DePRO study demonstrates the feasibility of a fully digital authentication and data-capture workflow in a population of patients with T2DM, with a high rate of completion of questionnaires by participants. It also shows that 2D matrix codes on outer packages of medications can serve as a direct channel to patients. This approach enables researchers to collect PROs without the involvement of health care professionals. Further research is needed to optimize recruitment via the medication tracker app, pair data-capturing activities with valuable services for patients, and establish whether such remote recruitment can provide a suitable alternative to personal invitations, particularly in the context of German legislation that will allow patients to voluntarily make the data in their electronic health records available to researchers starting in 2023 [20].

## Appendix

Multimedia Appendix 1 Responses to the comorbidity questionnaire.

Multimedia Appendix 2 Patient’s rating of treatment satisfaction, convenience, and flexibility.

## Abbreviations

DTSQ: Diabetes Treatment Satisfaction Questionnaire
PRO: patient-reported outcome
SDSCA-G: German version of the Summary of Diabetes Self-Care Activities measure
T2DM: type 2 diabetes mellitus
