# Supplementary material for: Evaluation of Self-care Activities and Quality of Life in Patients With Type 2 Diabetes Treated With Metformin Using the 2D Matrix Code of Outer Drug Packages as Patient Identifier: the DePRO Proof-of-Concept Observational Study
Source: JMIR Diabetes. 2022 May 24;7(2):e31832. doi: 10.2196/31832 (PMC9175102; doi:10.2196/31832)
Supplement: Multimedia Appendix 1 [file diabetes_v7i2e31832_app1.docx]

# Multimedia Appendix 1

**Multimedia Appendix 1.** Responses to the comorbidity questionnaire.

|  | | | **Total** | | **No** | | **Yes** | |
| --- | --- | --- | --- | --- | --- | --- | --- | --- |
|  |  |  | **N** | **%** | **n** | **%** | **n** | **%** |
| Have you ever been to an ophthalmologist? | | | 29 | 100 | 2 | 7 | 27 | 93 |
|  | [For patients who responded “Yes”:] | |  |  |  |  |  |  |
|  |  | Did your ophthalmologist tell you, you have changes in the retina/fundus of your eye caused by diabetes? | 27 | 100 | 26 | 96 | 1 | 4 |
|  |  | Have you ever had laser eye surgery or received intraocular injections of medications in your eye? | 27 | 100 | 26 | 96 | 1 | 4 |
| Has your physician told you you have nerve damage (neuropathy) caused by diabetes? | | | 29 | 100 | 24 | 83 | 5 | 17 |
| Do you have symptoms such as tingling, numbness, burning or shooting pains in your feet/legs, especially in the evening when resting? | | | 29 | 100 | 22 | 76 | 7 | 24 |
| Was vibration sense tested on your feet using a tuning fork? | | | 29 | 100 | 12 | 41 | 17 | 59 |
| Have you ever had poorly healing wounds on your feet? | | | 29 | 100 | 28 | 97 | 1 | 3 |
| Has you general practitioner or diabetologist ever told you that your kidney is affected by diabetes or that you have impaired kidney function? | | | 29 | 100 | 25 | 86 | 4 | 14 |
| Has your general practitioner or diabetologist ever told you that you excrete more protein or albumin in your urine? | | | 29 | 100 | 25 | 86 | 4 | 14 |
| Have you ever had a heart attack, or have you had a stent or cardiac bypass surgery? | | | 29 | 100 | 26 | 90 | 3 | 10 |
| Have you ever had a stroke or temporary stroke (TIA) or have you had surgery on the cervical arteries? | | | 29 | 100 | 29 | 100 | 0 | 0 |
| Have you had your toes, feet or legs amputated? | | | 29 | 100 | 28 | 97 | 1 | 3 |
