# Supplementary material for: Evaluation of Self-care Activities and Quality of Life in Patients With Type 2 Diabetes Treated With Metformin Using the 2D Matrix Code of Outer Drug Packages as Patient Identifier: the DePRO Proof-of-Concept Observational Study
Source: JMIR Diabetes. 2022 May 24;7(2):e31832. doi: 10.2196/31832 (PMC9175102; doi:10.2196/31832)
Supplement: Multimedia Appendix 2 [file diabetes_v7i2e31832_app2.docx]

# Multimedia Appendix 2

**Multimedia Appendix 2**. Patient’s rating of treatment satisfaction, convenience, and flexibility (N=29).

|  | **Satisfaction with current treatment** | | **Convenience of the treatment** | | **Flexibility of the treatment** | | **Satisfaction with own understanding of their diabetes** | | **How satisfied to continue the present treatment** | |
| --- | --- | --- | --- | --- | --- | --- | --- | --- | --- | --- |
|  | **n** | **%** | **n** | **%** | **n** | **%** | **n** | **%** | **n** | **%** |
| **Missing** | 1 | 3 | 1 | 3 | 1 | 3 | 1 | 3 | 1 | 3 |
| **Extremely dissatisfied/‌inconvenient/‌inflexible** | 1 | 3 | 0 | 0 | 1 | 3 | 0 | 0 | 1 | 3 |
| **Very dissatisfied/‌inconvenient/‌inflexible** | 1 | 3 | 1 | 3 | 2 | 7 | 1 | 3 | 0 | 0 |
| **Dissatisfied/‌inconvenient/‌inflexible** | 1 | 3 | 2 | 7 | 1 | 3 | 2 | 7 | 0 | 0 |
| **Somewhat satisfied/‌convenient/‌flexible** | 1 | 3 | 2 | 7 | 3 | 10 | 3 | 10 | 3 | 10 |
| **Satisfied/‌convenient/‌flexible** | 6 | 21 | 5 | 17 | 3 | 10 | 4 | 14 | 2 | 7 |
| **Very satisfied/‌convenient/‌flexible** | 8 | 28 | 8 | 28 | 8 | 28 | 7 | 24 | 7 | 24 |
| **Extremely satisfied/‌convenient/‌flexible** | 10 | 35 | 10 | 35 | 10 | 35 | 11 | 38 | 15 | 52 |
